# Association between CHADS_2_ score, depressive symptoms, and quality of life in a general population

**DOI:** 10.1186/s12888-017-1214-5

**Published:** 2017-02-27

**Authors:** Guo-Zhe Sun, Ning Ye, Nai-Jin Zhang, Yuan Li, Shuang Chen, Ye Chang, Zhao Li, Ying-Xian Sun

**Affiliations:** Department of Cardiovascular Medicine, The First Hospital of China Medical University, 155 Nanjing Street, Heping, Shenyang, 110001 Liaoning China

**Keywords:** CHADS_2_ score, Patient Health Questionnaire-9, World Health Organization Quality of Life Brief Scale, Depressive symptoms, Quality of life

## Abstract

**Background:**

To investigate the association between CHADS_2_ score, depressive symptoms, and quality of life in a large general population from China.

**Methods:**

A cross-sectional study of 11,956 permanent residents of Liaoning Province in China ≥ 35 years of age was conducted between January and August 2013 (response rate 85.3%). All participants completed a questionnaire, had a physical examination, and underwent blood examination. Depressive symptoms were assessed with the Patient Health Questionnaire-9 (PHQ-9), while the quality of life (QoL) was measured using the World Health Organization Quality of Life Brief Scale (WHOQOL-BREF).

**Results:**

With increasing CHADS_2_ score, the prevalence of depressive symptoms increased from 4.9 to 27.8% (*P* < 0.001), and all scores of WHOQOL-BREF decreased significantly (all *P*s < 0.001). After adjusting for confounding risk factors, subjects with CHADS_2_ score ≥ 3 had higher risk of depressive symptoms than those with CHADS_2_ score = 0 (all *P*s < 0.05). Also, CHADS_2_ score was negatively associated with all scores of WHOQOL-BREF (all *P*s < 0.001). Furthermore, subjects with any item in CHADS_2_ had higher prevalence of depressive symptoms (all *P*s < 0.001). Heart failure and stroke remained independently associated with depressive symptoms after adjusting for confounding risk factors and other items (*P*s < 0.001), while heart failure, age ≥ 75 years, diabetes mellitus, and stroke were all independently negatively associated with the total score of WHOQOL-BREF (all *P*s < 0.05).

**Conclusions:**

The CHADS_2_ score is significantly associated with depressive symptoms and impaired quality of life in the general population.

## Background

Depression is a worldwide public health problem [1, 2], and depressive disorder has become one of the leading causes of worldwide disability [3], which could contribute to an increased risk of all-cause mortality [4]. High prevalence of depression has been reported in patients with cardiovascular diseases (CVD) and its presence increases the risk for adverse cardiovascular events [5]. However, the awareness of depressive symptoms among such population is quite low and the majority of them are not appropriately treated and controlled. Therefore, identifying all the epidemiological characteristics for depressive symptoms will help to create population-based strategies to prevent and treat this serious health problem.

In recent years, the measurement of health has been broadened beyond traditional health indicators such as mortality and morbidity. Thus, quality of life (QoL) is defined by the World Health Organization (WHO) as individuals’ perceptions of their position in life in the context of the culture and value systems in which they live and in relation to their goals, expectations, standards and concerns [6]. It has received growing attention recently, and can be impaired in patients with established CVD [7] or in individuals at high risk of CVD [8].

The CHADS_2_ score is a simple but reliable clinical scheme, which includes congestive heart failure, hypertension, age ≥ 75 years, diabetes mellitus, and prior stroke or transient ischemic attack. It has been widely used to assess the risk of stroke for patients with atrial fibrillation (AF) [9, 10]. Moreover, the application of the CHADS_2_ score was not limited in AF population or assessing the risk of stroke in recent studies. In patients with acute coronary syndrome with or without AF, the score could be used as a risk assessment tool for mortality [11]. Furthermore, the score is newly found to be associated with peripheral arterial occlusive disease in patients without AF [12].

However, the association of the CHADS_2_ score with depressive symptoms and QoL has never been reported, even though the prevalence of depression has been proved to be apparently higher in heart failure [13], hypertension [14], diabetes [15], and stroke [16] than in the general population [17]. Therefore, the current study was designed to explore the association between the CHADS_2_ score, depressive symptoms, and QoL in a general Chinese population.

## Methods

### Study population

A representative sample of men and women ≥ 35 years of age from Liaoning Province was recruited between January 2013 and August 2013, using a multi-stage, randomly stratified, cluster-sampling scheme, which is called the Northeast China Rural Cardiovascular Health Study (NCRCHS). And our current study about CHADS_2_ score, depressive symptoms, and quality of life was part of the NCRCHS. Three counties (Dawa, Zhangwu, and Liaoyang) were selected from the eastern, southern, and northern regions of Liaoning Province. One township near a city in each county was randomly selected giving a total of three townships. Six to eight villages from each township were randomly selected to give a total of 26 rural villages. All of the eligible permanent residents aged ≥ 35 years from each village (*n* = 14,016) were invited to participate in the study, and 11,956 (response rate 85.3%) agreed to do so. Participants with pregnancy and malignant tumor were excluded from the present study. And subjects with severe mental disorders (for example psychosis) who couldn’t complete the face-to-face survey of NCRCHS were also excluded.

The study was approved by the Ethics Committee of China Medical University (Shenyang, China). All procedures were performed in accordance with the ethical standards. Written consent was obtained from all participants after they had been informed of the study’s objectives, benefits, medical procedures, and confidentiality safeguards for personal information. In the case of an illiterate participant, written informed consent was obtained from the appropriate legal proxy.

### Data collection and measurement

Data were collected during a single clinic visit by doctors and trained nurses using a standard questionnaire in a face-to-face interview. All potential investigators had received training on the objectives of the study, how to administer the questionnaire, the standard methods of measurement, the importance of standardization, and study procedures. Only those who earned a perfect score on a post-training test were allowed to participate as study investigators. During data collection, the inspectors received further instructions and support.

Data on the demographic characteristics, lifestyle risk factors, family income, medical history of hypertension, congestive heart failure, stroke, diabetes mellitus, depressive symptoms, and quality of life were obtained, as described above, by interview with the standardized questionnaire. There was a central steering committee with a subcommittee for quality control that made sure all data were collected according to well-known standards.

### Depressive symptoms

Depressive symptoms were assessed using the Patient Health Questionnaire-9 (PHQ-9), which was a 9-item screening instrument and widely used in primary health centers with high reliability and validity [18, 19]. Participants were asked how often, over the past 2 weeks, they had been bothered by each of the depressive symptoms with the score ranging from 0 to 3. Then, the total score of PHQ-9 ranged from 0 to 27. The severity of depressive disorder was considered mild for score of 5 to 9, moderate for score of 10 to 14, moderately severe for score of 15 to 19 and severe for score of 20 to 27. A PHQ-9 score ≥ 10 was recommended as the cut-off score for detecting major depressive disorders [20].

### Quality of life

The quality of life was measured with the World Health Organization Quality of Life Brief Scale (WHOQOL-BREF), which was a self-report inventory with 26 original items [21, 22]. The items fell into four domains: the physical health (7 items), the psychological health (6 items), the social relationships (3 items), and the environment (8 items), together with 2 items measuring the overall QoL and general health. Each item was answered on a 5-point response scale, and the range of score for each domain was from 4 to 20 after calculation, with higher score indicating better QoL.

### CHADS_2_ score

The CHADS_2_ score was calculated for each subject based on the point system with 2 points for a history of stroke and 1 point for congestive heart failure, hypertension, an age ≥ 75 years and diabetes mellitus. Congestive heart failure was assigned positive if they had ever been told by a physician that they had congestive heart failure [23]. Similarly, stroke was defined as a history of prior stroke or transient ischemic attack. Hypertension was defined as a systolic blood pressure (SBP) ≥ 140 mmHg and/or diastolic blood pressure (DBP) ≥ 90 mmHg and/or the use of antihypertensive medications according to the Eighth Joint National Committee on the guidelines for the management of high blood pressure in adults (JNC-8) [24]. World Health Organization criteria were followed for defining diabetes mellitus (fasting blood glucose ≥ 7.0 mmol/L or 126 mg/dL, and/or being on treatment for diabetes) [25].

### Covariate measurements

Weight and height were measured to the nearest 0.1 kg and 0.1 cm, respectively, with the participants in lightweight clothing without shoes. The body mass index (BMI) was calculated as weight in kilograms divided by the square of the height in meters.

Fasting blood samples were collected in the morning after ≥ 8 h of fasting for all participants. Blood samples were obtained from an antecubital vein using BD Vacutainer tubes containing EDTA. Serum was subsequently isolated from whole blood, and all serum samples were frozen at −20 °C for testing at a central, certified laboratory. Fasting blood glucose (FBG), total cholesterol (TC), triglycerides (TG), high density lipid cholesterol (HDL-C), low density lipid cholesterol (LDL-C), serum uric acid, serum creatinine, and other routine blood indices were analyzed enzymatically on an auto-Analyzer. Glomerular filtration rate (GFR) was estimated using the equation originating from the Chronic Kidney Disease Epidemiology Collaboration (CKD-EPI) equation [26].

Physical activity included occupational and leisure-time physical activity, and a detailed description of these standards has been presented [27]. The combination of occupational and leisure-time physical activity was therefore described as low (light levels of activity in both categories), moderate (moderate or high levels of activity in one of the categories), or high (moderate or high levels of activity in both categories).

### Statistical analysis

All statistical analyses were performed using SPSS 17.0 software (SPSS Inc., Chicago, IL, USA). Differences between groups were compared using two-tailed Student’s *t*-test, ANOVA or the *χ* ^2^ test as appropriate. The prevalences of depressive symptoms among different CHADS_2_ score were calculated, and the total score of WHOQOL-BREF by PHQ-9 score and CHADS_2_ score was also presented. Both univariate and multivariate logistic regression analyses were performed to estimate the crude and independent associations between each item in CHADS_2_, CHADS_2_ score, and depressive symptoms. Multivariate linear regression analysis and optimal scale regression analysis were conducted to assess the independent associations of each item in CHADS_2_ and CHADS_2_ score with WHOQOL-BREF score, respectively. *P* < 0.05 was considered as statistically significant.

## Results

### Characteristics of the study population

Of the 11,956 participants, 896 had incomplete data and were excluded from the analysis, leaving a total of 11,060 participants (5080 men and 5980 women) with a mean age of 53.9 years. The subjects with moderate or severe depressive symptoms were significantly older and had lower percentage of men than those with no or mild depressive symptoms (*P*s < 0.001) (Table 1). The subjects with moderate or severe depressive symptoms had significantly higher FBG, TG (all *P*s < 0.05), and lower eGFR levels (*P* < 0.001). They also had a lower percentage of alcohol drinking and smoking and lower levels of education, income, physical activity and sleep time (all *P*s < 0.001). In addition, the subjects with moderate or severe depressive symptoms had significantly higher levels of CHADS_2_ score and lower scores of WHOQOL-BREF (all *P*s < 0.001). However, there were no significant differences in BMI, SBP, DBP, TC, HDL-C, LDL-C, or serum uric acid between the two groups.

**Table 1:** Comparison of the characteristics of the study sample

Variable	All (*n* = 11,060)	Depressive symptoms		*P* value
		No or mild (*n* = 10,390)	Moderate or severe (*n* = 670)	
Age, years	53.9 ± 10.5	53.7 ± 10.5	57.1 ± 10.2	<0.001
Male	5080 (45.9)	4898 (47.1)	182 (27.2)	<0.001
BMI, kg/m^2^	24.8 ± 3.7	24.8 ± 3.7	24.5 ± 3.8	0.050
SBP, mmHg	141.6 ± 23.3	141.5 ± 23.2	143.5 ± 25.5	0.051
DBP, mmHg	82.0 ± 11.7	82.0 ± 11.7	82.2 ± 11.8	0.603
FBG, mmol/L	5.91 ± 1.62	5.90 ± 1.61	6.09 ± 1.76	0.006
TC, mmol/L	5.24 ± 1.09	5.23 ± 1.08	5.31 ± 1.18	0.124
TG, mmol/L	1.63 ± 1.47	1.62 ± 1.47	1.75 ± 1.51	0.030
HDL-C, mmol/L	1.41 ± 0.38	1.41 ± 0.38	1.40 ± 0.40	0.601
LDL-C, mmol/L	2.93 ± 0.82	2.92 ± 0.82	2.96 ± 0.89	0.290
Serum uric acid, umol/L	292 ± 85	292 ± 85	289 ± 90	0.344
eGFR, ml/min/1.73 m^2^	92.8 ± 15.9	93.1 ± 15.7	88.6 ± 18.3	<0.001
Current smoker	3886 (35.1)	3693 (35.5)	193 (28.8)	<0.001
Current drinker	2474 (22.4)	2402 (23.1)	72 (10.7)	<0.001
Education level				<0.001
≤ Primary school	5539 (50.1)	5095 (49.0)	444 (66.3)	
Middle school	4491 (40.6)	4296 (41.3)	195 (29.1)	
≥ High school	1030 (9.3)	999 (9.6)	31 (4.6)	
Family income, CNY/year				<0.001
≤ 5000	1379 (12.5)	1209 (11.6)	170 (25.4)	
5000–20,000	6025 (54.5)	5655 (54.4)	370 (55.2)	
> 20,000	3656 (33.1)	3526 (33.9)	130 (19.4)	
Physical activity				<0.001
Low	3263 (29.5)	2953 (28.4)	310 (46.3)	
Moderate	7164 (64.8)	6830 (65.7)	334 (49.9)	
High	633 (5.7)	607 (5.8)	26 (3.9)	
Sleep duration, h/day	7.3 ± 1.7	7.3 ± 1.6	6.2 ± 2.1	<0.001
Congestive heart failure	102 (0.9)	78 (0.8)	24 (3.6)	<0.001
Hypertension	5623 (50.8)	5234 (50.4)	389 (58.1)	<0.001
Age ≥ 75 years	332 (3.0)	297 (2.9)	35 (5.2)	0.001
Diabetes mellitus	1154 (10.4)	1051 (10.1)	103 (15.4)	<0.001
Stroke	976 (8.8)	848 (8.2)	128 (19.1)	<0.001
CHADS_2_ score	0.83 ± 0.96	0.80 ± 0.94	1.20 ± 1.25	<0.001
WHOQOL-BREF domain				
Physical health	15.1 ± 2.3	15.4 ± 2.1	11.5 ± 2.5	<0.001
Psychological	14.5 ± 2.4	14.8 ± 2.2	10.7 ± 2.6	<0.001
Social relationships	14.6 ± 2.1	14.7 ± 2.0	13.0 ± 2.4	<0.001
Environment	13.5 ± 2.1	13.6 ± 2.1	11.7 ± 2.1	<0.001

Data are expressed as mean ± standard deviation or *n* (%)

*Abbreviations*: *BMI* body mass index, *CNY* China Yuan, *DBP* diastolic blood pressure, *FBG* fasting blood glucose, *eGFR* estimated glomerular filtration rate, *HDL-C* high density lipid cholesterol, *LDL-C* low density lipid cholesterol, *SBP* systolic blood pressure, *TC* total cholesterol, *TG* triglycerides, *WHOQOL-BREF* the World Health Organization Quality of Life Brief Scale

### Prevalence of depressive symptoms and QoL by CHADS_2_ score

**Fig. 1:**
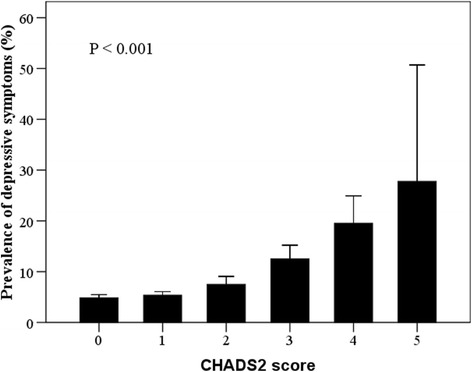
Prevalence of depressive symptoms by *CHADS*_*2*_ *score*. Percentage of subjects with a PHQ-9 score ≥ 10 increases with increasing CHADS_2_ score (*P* < 0.001). *Error bars* represent standard deviation. PHQ-9: Patient Health Questionnaire-9

**Fig. 2:**
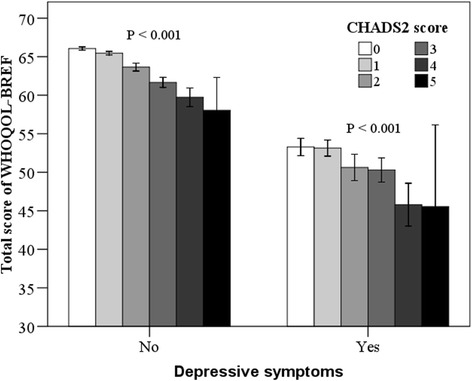
Total score of WHOQOL-BREF by CHADS_2_ score stratified by depressive symptoms. The total score of *WHOQOL-BREF decreases* siginificantly with *increasing CHADS*_*2*_ *score* in both subgroups (*P*s < 0.001). *Error bars* represent standard deviation. PHQ-9: Patient Health Questionnaire-9; WHOQOL-BREF: World Health Organization Quality of Life Brief Scale

The prevalence of depressive symptoms (PHQ-9 score ≥ 10) by CHADS_2_ score was summarized (Fig. 1 and Table 2). It was found that the percentage of subjects with depressive symptoms increased significantly with increasing CHADS_2_ score, from the lowest of 4.9% to the highest of 27.8% (*P* < 0.001). The QoL evaluated by WHOQOL-BREF was described according to CHADS_2_ score (Table 3). As a result, the mean scores for total and every domain of WHOQOL-BREF decreased significantly with increasing CHADS_2_ score (all *P*s < 0.001). The association of CHADS_2_ score with the total score of WHOQOL-BREF stratified by depressive symptoms was also examined and presented (Fig. 2). The total score of WHOQOL-BREF decreased siginificantly with increasing CHADS_2_ score in both subgroups (*P*s < 0.001). The lowest level of total score was found in subjects with CHADS_2_ score of 0, while the highest was in those with CHADS_2_ score of 5.

**Table 2:** Logistic regression analyses for associations between CHADS_2_ score and depressive symptoms (PHQ-9 score ≥ 10)

	Total (*n*)	Depressive symptoms *n* (%)	Unadjusted model		Adjusted model 1		Adjusted model 2	
			OR (95% CI)	*P* value	OR (95% CI)	*P* value	OR (95% CI)	*P* value
Congestive heart failure								
No	10,958	646 (5.9)	1		1		1	
Yes	102	24 (23.5)	4.91 (3.09–7.81)	<0.001	3.17 (1.91–5.25)	<0.001	2.98 (1.79–4.96)	<0.001
Hypertension								
No	5437	281 (5.2)	1		1		1	
Yes	5623	389 (6.9)	1.36 (1.16–1.60)	<0.001	1.15 (0.96–1.37)	0.127	1.04 (0.87–1.24)	0.687
Age ≥ 75 years								
No	10,728	635 (5.9)	1		1		1	
Yes	332	35 (10.5)	1.87 (1.31–2.68)	0.001	1.01 (0.68–1.51)	0.957	1.04 (0.70–1.55)	0.851
Diabetes mellitus								
No	9906	567 (5.7)	1		1		1	
Yes	1154	103 (8.9)	1.61 (1.30–2.01)	<0.001	1.35 (1.07–1.72)	0.013	1.25 (0.98–1.59)	0.076
Stroke								
No	10,084	542 (5.4)	1		1		1	
Yes	976	128 (13.1)	2.66 (2.17–3.26)	<0.001	2.02 (1.62–2.53)	<0.001	1.96 (1.56–2.46)	<0.001
CHADS_2_ score								
0	4888	238 (4.9)	1		1			
1	4253	229 (5.4)	1.11 (0.92–1.34)	0.265	1.02 (0.83–1.24)	0.872		
2	1093	82 (7.5)	1.59 (1.22–2.06)	0.001	1.15 (0.87–1.53)	0.329		
3	598	75 (12.5)	2.80 (2.13–3.69)	<0.001	1.99 (1.47–2.68)	<0.001		
4	210	41 (19.5)	4.74 (3.29–6.83)	<0.001	3.47 (2.29–5.25)	<0.001		
5	18	5 (27.8)	7.52 (2.66–21.25)	<0.001	3.94 (1.28–12.12)	0.017		

Adjusted model 1: adjusted for gender, body mass index, total cholesterol, triglyceride, low density lipid cholesterol, high density lipid cholesterol, serum uric acid, estimated glomerular filtration rate, smoking, drinking, education, income, physical activity, and sleep time; Adjusted model 2: adjusted for factors in model 1 and other items in CHADS_2_

*Abbreviations*: *CI* confidence interval, *OR* odds ratio, *PHQ-9* Patient Health Questionnaire-9

**Table 3:** The mean score of WHOQOL-BREF based on CHADS_2_ score

	CHADS_2_ score						*P* value
	0 (*n* = 4888)	1 (*n* = 4253)	2 (*n* = 1093)	3 (*n* = 598)	4 (*n* = 210)	5 (*n* = 18)	
Overall Quality of life	3.30 ± 0.70	3.28 ± 0.68	3.23 ± 0.73	3.15 ± 0.63	3.02 ± 0.72	2.94 ± 0.64	<0.001
General health	3.49 ± 0.82	3.39 ± 0.83	3.24 ± 0.91	2.95 ± 0.89	2.64 ± 0.85	2.72 ± 1.02	<0.001
Domains							
Physical health	15.5 ± 2.1	15.2 ± 2.2	14.5 ± 2.5	13.7 ± 2.5	12.5 ± 2.9	11.6 ± 3.1	<0.001
Psychological	14.7 ± 2.4	14.7 ± 2.4	14.2 ± 2.5	13.6 ± 2.5	12.9 ± 3.0	12.2 ± 3.0	<0.001
Social relationships	14.9 ± 2.1	14.7 ± 2.0	14.2 ± 2.2	13.9 ± 2.1	13.2 ± 2.4	12.6 ± 2.1	<0.001
Environment	13.6 ± 2.1	13.5 ± 2.1	13.3 ± 2.2	13.0 ± 2.1	12.7 ± 2.3	12.5 ± 1.9	<0.001
Total Score	65.4 ± 8.2	64.8 ± 8.0	62.7 ± 8.9	60.2 ± 8.4	57.0 ± 9.8	54.6 ± 9.2	<0.001

Data are expressed as mean ± standard deviation

*Abbreviations*: *WHOQOL-BREF* the World Health Organization Quality of Life Brief Scale

### Association between CHADS_2_ score and depressive symptoms

The associations between each item in CHADS_2_, CHADS_2_ score and depressive symptoms were examined by univariate and multivariate logistic regression analyses (Table 2). Heart failure and stroke were independently associated with depressive symptoms after adjusting for other items in CHADS_2_ and clinical covariates, including gender, BMI, TC, TG, LDL-C, HDL-C, serum uric acid, eGFR, smoking, drinking, education, income, physical activity, and sleep time (*P*s < 0.001). However, the associations between hypertension, age ≥ 75 years, diabetes mellitus and depressive symptoms were significant only in univariate model (*P*s ≤ 0.001) but not in multivariate model (*P*s > 0.05). Subjects with CHADS_2_ score ≥ 3 had significantly higher independent risk of depressive symptoms than those with CHADS_2_ score = 0 after adjusting for clinical covariates (all *P*s < 0.05).

### Association between CHADS_2_ score and QoL

To explore the associations between CHADS_2_ score and QoL, a multivariate linear regression analysis was conducted for each item in CHADS_2_, and optimal scale regression analysis was used for CHADS_2_ score (as ordinal variate) (Table 4). Generally speaking, items in CHADS_2_ were negatively associated with scores of WHOQOL-BREF. Congestive heart failure, age ≥ 75 years, diabetes mellitus, and stroke were all independently negatively associated with the total score of WHOQOL-BREF after adjusting for gender, BMI, TC, TG, LDL-C, HDL-C, serum uric acid, eGFR, smoking, drinking, education, income, physical activity, sleep time, and other items in CHADS_2_ (all *P*s < 0.05). Optimal scale regression analysis showed CHADS_2_ score was independently negatively associated with all scores of WHOQOL-BREF (all *P*s < 0.001).

**Table 4:** Multivariate linear and optimal scale regression analyses for associations between CHADS_2_ score and WHOQOL-BREF

	Overall quality of life	General health	Domains				Total score
			Physical health	Psychological	Social relationships	Environment	
Congestive heart failure^a^							
β	−0.106	−0.482	−1.771	−1.092	−0.528	−0.482	−4.458
*P* value	0.113	<0.001	<0.001	<0.001	0.008	0.016	<0.001
Hypertension^a^							
β	0.006	−0.073	−0.154	0.061	−0.097	0.075	−0.169
*P* value	0.690	<0.001	<0.001	0.193	0.018	0.071	0.284
Age ≥ 75 years^a^							
β	0.115	0.230	−0.492	−0.070	−0.581	−0.093	−0.877
*P* value	0.003	<0.001	<0.001	0.596	<0.001	0.426	0.047
Diabetes mellitus^a^							
β	−0.039	−0.190	−0.368	−0.307	−0.160	−0.132	−1.198
*P* value	0.191	<0.001	<0.001	0.003	0.072	0.145	<0.001
Stroke^a^							
β	−0.091	−0.377	−1.192	−0.809	−0.469	−0.384	−3.298
*P* value	<0.001	<0.001	<0.001	<0.001	<0.001	<0.001	<0.001
CHADS_2_ score^b^							
β	−0.045	−0.170	−0.180	−0.098	−0.098	−0.058	−0.130
*P* value	<0.001	<0.001	<0.001	<0.001	<0.001	<0.001	<0.001

*Abbreviations*: *CI* confidence interval, *WHOQOL-BREF* the World Health Organization Quality of Life Brief Scale

^a^by multivariate linear regression analysis adjusted for gender, body mass index, total cholesterol, triglyceride, low density lipid cholesterol, high density lipid cholesterol, serum uric acid, estimated glomerular filtration rate, smoking, drinking, education, income, physical activity, sleep time, and other items in CHADS_2_

^b^by optimal scale regression analysis adjusted for all factors in model α except for items in CHADS_2_

## Discussion

The results of this study demonstrated that the CHADS_2_ score is significantly associated with depressive symptoms and QoL in a large general population. The tools of WHOQOL-BREF and PHQ-9 were chosen to evaluate QoL and depressive symptoms, respectively. With increasing CHADS_2_ score, the prevalence of depressive symptoms (PHQ-9 score ≥ 10) increases, while the total score and every domain of WHOQOL-BREF decreases. The independent associations were still observed after adjusting for confounding risk factors.

Previous studies demonstrated that the components of CHADS_2_ score were associated with depression and poor QoL. Depression in patients with heart failure was 4–5 times that of the general population [28], and QoL was greatly impaired among patients with heart failure [29]. A systemic review showed that the general prevalence of depression was as high as 26.8% among hypertensive patients [14], and hypertension also impaired QoL [30]. Diabetes had a marked increase in depression prevalence, however, a reduction in QoL [31]. Moreover, depression was a common and serious complication after stroke, affecting nearly 30% of patients at different stages [32]. The possible reasons for lower QoL and depressive symptoms among these patients included having chronic diseases, being aware of them, and comprehensive treatment [33]. In this current study, only heart failure and stroke were independently associated with depressive symptoms, while old age, hypertension, and diabetes mellitus had no significant independent relation. All items in CHADS_2_ except for hypertension were associated with impaired QoL. These results were somewhat consistent with previous ones. Relatively low education level, awareness, and treatment of chronic diseases in rural China may partially contribute to the differences [34, 35].

The scheme of the CHADS_2_ score has been used widely, and a CHADS_2_ score ≥ 2 is considered as the risk factor for stroke in AF patients. Recent study showed that patients with a CHADS_2_ score ≥ 2 was also significantly associated with peripheral arterial occlusive disease among non-AF population [12]. The present study in the general population demonstrated that subjects with CHADS_2_ score ≥ 3 had significantly higher risk of depressive symptoms than those with CHADS_2_ score = 0 after adjusting for clinical covariates. Furthermore, CHADS_2_ score was negatively associated with all scores of WHOQOL-BREF. Previous study showed that depression was accompanied by poor QoL with low scores of WHOQOL-BREF [36]. This study found that CHADS_2_ score was negatively associated with the total score of WHOQOL-BREF whether or not the subjects had depressive symptoms. This is the first study to reveal the associations of CHADS_2_ score with depressive symptoms and impaired QoL.

This study has several limitations. First, the current study is cross-sectional and it was not clear whether the CHADS_2_ score would predict the incidence of depressive symptoms. Second, congestive heart failure and stroke were diagnosed based on a previous history of physician-diagnosis since a large sample was involved. This approach may give rise to an unintentional bias though it had been utilized in previous studies [23, 37]. Third, the current study was part of NCRCHS, and subjects with severe mental disorders who couldn’t complete the survey were excluded. This may have a potential bias on the results. Fourth, the relatively small sample in some subgroups may have reduced the statistical power, and further studies with larger sample were needed to confirm the results.

## Conclusions

This is the first study to demonstrate that the CHADS_2_ score is significantly associated with depressive symptoms and impaired QoL in the general Chinese population. Much attention should also be paid to depressive symptoms and QoL among patients with high CHADS_2_ score.

## Abbreviations

AF: Atrial fibrillation
CI: Confidence interval
CVD: Cardiovascular diseases
DBP: Diastolic blood pressure
FBG: Fasting blood glucose
GFR: Glomerular filtration rate
HDL-C: High density lipid cholesterol
LDL-C: Low density lipid cholesterol
OR: Odds ratio
PHQ-9: Patient Health Questionnaire-9
QoL: Quality of life
SBP: Systolic blood pressure
TC: Total cholesterol
TG: Triglycerides
WHO: World Health Organization
WHOQOL-BREF: World Health Organization Quality of Life Brief Scale

## References

[1] Andrade L, Caraveo-Anduaga JJ, Berglund P, Bijl RV, De Graaf R, Vollebergh W (2003). The epidemiology of major depressive episodes: results from the international consortium of psychiatric epidemiology (ICPE) surveys. Int J Methods Psychiatr Res.

[2] Alonso J, Angermeyer MC, Bernert S, Bruffaerts R, Brugha TS, Bryson H (2004). Prevalence of mental disorders in Europe: results from the European Study of the Epidemiology of Mental Disorders (ESEMeD) project. Acta Psychiatr Scand Suppl.

[3] Murray CJ, Lopez AD (1997). Alternative projections of mortality and disability by cause 1990–2020: global burden of disease study. Lancet.

[4] Chesney E, Goodwin GM, Fazel S (2014). Risks of all-cause and suicide mortality in mental disorders: a meta-review. World Psychiatry.

[5] Whooley MA (2006). Depression and cardiovascular disease: healing the broken-hearted. JAMA.

[6] WHOQOL Group. The World Health Organization Quality of Life assessment (WHOQOL): position paper from the World Health Organization. Soc Sci Med. 1995;41:1403–9. 10.1016/0277-9536(95)00112-k 8560308

[7] Cepeda-Valery B, Cheong AP, Lee A, Yan BP (2011). Measuring health related quality of life in coronary heart disease: the importance of feeling well. Int J Cardiol.

[8] Ko HY, Lee JK, Shin JY, Jo E (2015). Health-related quality of life and cardiovascular disease risk in Korean adults. Korean J Fam Med.

[9] Gage BF, van Walraven C, Pearce L, Hart RG, Koudstaal PJ, Boode BS (2004). Selecting patients with atrial fibrillation for anticoagulation: stroke risk stratification in patients taking aspirin. Circulation.

[10] Gage BF, Waterman AD, Shannon W, Boechler M, Rich MW, Radford MJ (2001). Validation of clinical classification schemes for predicting stroke: results from the national registry of atrial fibrillation. JAMA.

[11] Poçi D, Hartford M, Karlsson T, Herlitz J, Edvardsson N, Caidahl K (2012). Role of the CHADS_2_ score in acute coronary syndromes: risk of subsequent death or stroke in patients with and without atrial fibrillation. Chest.

[12] Hsu PC, Lin TH, Lee WH, Chu CY, Chiu CA, Lee HH (2014). Association between the CHADS_2_ score and an ankle-brachial index of < 0.9 in patients without atrial fibrillation. J Atheroscler Thromb.

[13] Rutledge T, Reis VA, Linke SE, Greenberg BH, Mills PJ (2006). Depression in heart failure a meta-analytic review of prevalence, intervention effects, and associations with clinical outcomes. J Am Coll Cardiol.

[14] Li Z, Li Y, Chen L, Chen P, Hu Y (2015). Prevalence of depression in patients with hypertension: a systematic review and meta-analysis. Medicine (Baltimore).

[15] Anderson RJ, Freedland KE, Clouse RE, Lustman PJ (2001). The prevalence of comorbid depression in adults with diabetes: a meta-analysis. Diabetes Care.

[16] Fei K, Benn EK, Negron R, Arniella G, Tuhrim S, Horowitz CR (2016). Prevalence of depression among stroke survivors: racial-ethnic differences. Stroke.

[17] Zhou X, Bi B, Zheng L, Li Z, Yang H, Song H (2014). The prevalence and risk factors for depression symptoms in a rural Chinese sample population. Plos One.

[18] Löwe B, Kroenke K, Herzog W, Gräfe K (2004). Measuring depression outcome with a brief self-report instrument: sensitivity to change of the patient health questionnaire (PHQ-9). J Affect Disord.

[19] Kroenke K, Spitzer RL, Williams JB (2001). The PHQ-9: validity of a brief depression severity measure. J Gen Intern Med.

[20] Manea L, Gilbody S, Mcmillan D (2012). Optimal cut-off score for diagnosing depression with the patient health questionnaire (PHQ-9): a meta-analysis. CMAJ.

[21] The WHOQOL Group (1998). Development of the World Health Organization WHOQOL-BREF quality of life assessment. Psychol Med.

[22] Skevington SM, Lotfy M, O’Connell KA, WHOQOL Group (2004). The World Health Organization’s WHOQOL-BREF quality of life assessment: psychometric properties and results of the international field trial. A report from the WHOQOL group. Qual Life Res.

[23] Chao TF, Lin YJ, Tsao HM, Tsai CF, Lin WS, Chang SL (2011). CHADS_2_ and CHA_2_DS_2_-VASc scores in the prediction of clinical outcomes in patients with atrial fibrillation after catheter ablation. J Am Coll Cardiol.

[24] James PA, Oparil S, Carter BL, Cushman WC, Dennison-Himmelfarb C, Handler J (2014). 2014 evidence-based guideline for the management of high blood pressure in adults: report from the panel members appointed to the Eighth Joint National Committee (JNC 8). JAMA.

[25] World Health Organization and International Diabetes Fedaration (2006). Definition and diagnosis of diabetes mellitus and intermediate hyperglycemia: report of a WHO/IDF consultation.

[26] Levey AS, Stevens LA, Schmid CH, Zhang YL, Castro AF, Feldman HI (2009). A new equation to estimate glomerular filtration rate. Ann Intern Med.

[27] Hu G, Tuomilehto J, Silventoinen K, Barengo N, Jousilahti P (2004). Joint effects of physical activity, body mass index, waist circumference and waist-to-hip ratio with the risk of cardiovascular disease among middle-aged finnish men and women. Eur Heart J.

[28] Khan S, Khan A, Ghaffar R, Awan ZA (2012). Frequency of depression in patients with chronic heart failure. J Ayub Med Coll Abbottabad.

[29] Berry C, Mcmurray J (1999). A review of quality-of-life evaluations in patients with congestive heart failure. Pharmacoeconomics.

[30] Carvalho MV, Siqueira LB, Sousa AL, Jardim PC (2013). The influence of hypertension on quality of life. Arq Bras Cardiol.

[31] Mishra SR, Sharma A, Bhandari PM, Bhochhibhoya S, Thapa K (2015). Depression and health-related quality of life among patients with type 2 diabetes mellitus: a cross-sectional study in Nepal. Plos One.

[32] Paolucci S (2008). Epidemiology and treatment of post-stroke depression. Neuropsychiatr Dis Treat.

[33] Hayes DK, Denny CH, Keenan NL, Croft JB, Greenlund KJ (2008). Health-related quality of life and hypertension status, awareness, treatment, and control: National Health and Nutrition Examination Survey, 2001–2004. J Hypertens.

[34] Bundy JD, He J (2016). Hypertension and related cardiovascular disease burden in China. Ann Glob Health.

[35] Liu X, Li Y, Li L, Zhang L, Ren Y, Zhou H (2016). Prevalence, awareness, treatment, control of type 2 diabetes mellitus and risk factors in Chinese rural population: the RuralDiab study. Sci Rep.

[36] Chang YC, Yao G, Hu SC, Wang JD (2015). Depression affects the scores of all facets of the WHOQOL-BREF and may mediate the effects of physical disability among community-dwelling older adults. Plos One.

[37] Schocken DD, Arrieta MI, Leaverton PE, Ross EA (1992). Prevalence and mortality rate of congestive heart failure in the United States. J Am Coll Cardiol.

